# HIV-1 selectively exploits cross-reactive CTL “help” to promote dysfunctional programming of pro-inflammatory dendritic cells

**DOI:** 10.1186/1742-4690-9-S2-P282

**Published:** 2012-09-13

**Authors:** RB Mailliard, KN Smith, RJ Fecek, CR Zaccard, SC Watkins, G Rappocciolo, JI Mullins, CR Rinaldo

**Affiliations:** 1 University of Pittsburgh, Pittsburgh, PA, USA; 2 University of Washington, Seattle, WA, USA

## Background

The ability of HIV-1 to rapidly accumulate mutations provides the virus with an effective means of escaping CD8+ cytotoxic T lymphocyte (CTL) responses. Here we describe how subtle alterations in CTL epitopes expressed by naturally occurring HIV-1 variants can result in an incomplete escape from pre-existing CTL recognition to create a pro-inflammatory environment, providing the virus with a selective advantage.

## Methods

We developed a dendritic cell (DC)-based in vitro model to induce primary CTLs specific against naturally occuring HIV-1 Gag epitope variants identified through sequencing of virus obtained in a longitudunal study from a subject with chronic HIV-1 infection. CTL function was assessed by ELISPOT, flow cytometry, and 51Cr-release cytoxicity assays.

## Results

The HIV-1 specific CTLs generated proved to be broadly cross-reactive. While the magnitudes of the responses between the variant and priming peptides were often similar, important qualitative differences were found. Most notably, epitope variants preferentially induced the "helper" activity of the CTLs while inhibiting their killing capacity. Importantly, instead of eliminating variant antigen-expressing immature DC as a negative immune feedback mechanism, the cross-reactive CTLs promoted the differentiation of DC into highly reactive mature DC capable of producing enhanced levels of IL-12 and IL-6, as well as the T cell chemoattractants CXCL10 and CCL5. These CTL-matured DC also develped long interconnected nanotubule extensions capable of facilitating intracellular transfer of HIV-1.

## Conclusion

The selective induction of pre-existing CTL "helper" activity in the absence of killing, induced by altered peptide presentation, adds a novel dimension to the concept of “original antigenic sin”. This phenomenon likely contributes to the chronic immune activation associated with HIV-1 infection and could be utilized by HIV-1 to promote spread and persistence. Developing a means to specifically interrupt the described CTL-DC positive immune feedback loop could prove critical for the effectiveness of future anti-HIV-1 vaccine therapies.

